# Are neuronal mechanisms of attention universal across human sensory and motor brain maps?

**DOI:** 10.3758/s13423-024-02495-3

**Published:** 2024-04-08

**Authors:** Edgar A. DeYoe, Wendy Huddleston, Adam S. Greenberg

**Affiliations:** 1 Department of Radiology, Medical College of Wisconsin, 8701 Watertown Plank Rd, Milwaukee, WI 53226 USA; 2 School of Rehabilitation Sciences and Technology, College of Health Professions and Sciences, University of Wisconsin - Milwaukee, 3409 N. Downer Ave, Milwaukee, WI 53211 USA; 3 Department of Biomedical Engineering, Medical College of Wisconsin and Marquette University, Milwaukee, WI 53226 USA; 4 Signal Mountain, USA

**Keywords:** Attention, Human, Motor, Vision, Audition, Cortical maps

## Abstract

One's experience of shifting attention from the color to the smell to the act of picking a flower seems like a unitary process applied, at will, to one modality after another. Yet, the unique and separable experiences of sight versus smell versus movement might suggest that the neural mechanisms of attention have been separately optimized to employ each modality to its greatest advantage. Moreover, addressing the issue of universality can be particularly difficult due to a paucity of existing cross-modal comparisons and a dearth of neurophysiological methods that can be applied equally well across disparate modalities. Here we outline some of the conceptual and methodological issues related to this problem and present an instructive example of an experimental approach that can be applied widely throughout the human brain to permit detailed, quantitative comparison of attentional mechanisms across modalities. The ultimate goal is to spur efforts across disciplines to provide a large and varied database of empirical observations that will either support the notion of a universal neural substrate for attention or more clearly identify the degree to which attentional mechanisms are specialized for each modality.

## A broad perspective

The first clear definition of attention is often ascribed to William James (James, 1890), who cast it as “the taking possession by the mind in clear and vivid form, of one out of what seem several simultaneously possible objects or trains of thought.” Perhaps it is this seemingly visual description that motivated subsequent studies of attention and its neural basis to focus heavily on the modality of vision (Beauchamp et al., 2001; Beck & Kastner, 2005; Boynton, 2005, 2009; Brefczynski & DeYoe, 1999; Bundesen, 1990; Buracas & Boynton, 2007; Buschman & Kastner, 2015; Carrasco, 2006, 2011; Carrasco et al., 2000; Carrasco & Yeshurun, 2009; Chou & Sen, 2021; Colby & Goldberg, 1999; Corbetta & Shulman, 2002; Culham et al., 1998; Datta & DeYoe, 2009; Desimone, 1998; Desimone & Duncan, 1995; DeYoe & Brefczynski, 2005; Huddleston & DeYoe, 2008; Itti & Koch, 2001; Kastner et al., 1998; Kastner & Ungerleider, 2000; Kelley et al., 2008; Lee & Maunsell, 2009; Lu & Dosher, 1998; Luck & Ford, 1998; Luck & Hillyard, 1995; Martinez-Trujillo & Treue, 2004; Maunsell & Treue, 2006; Maunsell, 2015; McMains & Somers, 2004; Moran & Desimone, 1985; Pessoa et al., 2003; Posner & Petersen, 1990; Puckett & DeYoe, 2015; Reynolds & Heeger, 2009; Roelfsema et al., 1998; Saygin & Sereno, 2008; Serences & Yantis, 2006; Shipp, 2004; Silver et al., 2005, 2007; Sperling et al., 2001; Szczepanski et al., 2010; Treisman & Gelade, 1980; Tsotsos, 2011; Wojciulik et al., 1998; Womelsdorf et al., 2008; Yantis & Serences, 2003). Yet, it is rarely mentioned that James also pointed out that attention influences the motor system as well, noting that “…reaction time is shorter when one concentrates his attention on the expected movement than when one concentrates it on the expected signal”(James, 1890). In accord with James, we suggest that it is opportune to ask if the considerable insights gained from the study of visual attention allow us to propose more general hypotheses about attention in the motor system as well as other non-visual sensory modalities. In vision, it is well established that focal attention is retinotopically specific (Brefczynski-Lewis et al., 2009; DeYoe & Brefczynski, 2005), and can be directed selectively to behaviorally relevant locations or objects in visual space (Cave & Bichot, 1999; Chen, 2012). Moreover, one can readily attend to a variety of other sensory features as well as eye and body movements (Beauchamp et al., 1997; Bisley & Goldberg, 2003; Clark et al., 2015; Da Costa et al., 2013; Greenberg et al., 2010; Greenberg & Gmeindl, 2008; Huddleston et al., 2008; Li et al., 2021; Puckett et al., 2017; Wulf & Prinz, 2001). From a phenomenological perspective, volitionally shifting attention from, say, the color of a flower to its smell to the movement needed to pick it feels like simply shifting a unitary attentional "spotlight" from one feature to another even though those features are in different modalities. In contrast, trying to attend to color, smell, and movement truly simultaneously (rather than very rapidly switching) seems difficult if not impossible. Does this experience imply a common, monolithic neural mechanism applied to all modalities, or are multiple, modality-specific attentional mechanisms just perceptually indistinguishable?

Our current understanding of the neural basis of attentional selection across modalities is unclear. At one extreme, neural mechanisms of attention may be shared across all modalities. Indeed, it has been proposed that "covert spatial attention emerges as a consequence of the reciprocal interactions between neural circuits primarily involved in specifying the visual properties of potential targets and those involved in specifying the movements needed to fixate them" (Moore et al., 2003). At the other extreme, one might suppose that, through evolution, attentional mechanisms may have been uniquely specialized for each modality causing attentional behavior to vary across them (Kong et al., 2014). To address this issue, we must carefully examine non-visual modalities to determine if they require a substantially different theoretical framework or if a common model applies to all.

To facilitate this endeavor, we begin by identifying certain aspects of attention-related neural function that might be fruitfully compared across modalities (see "Extending attention-related concepts across modalities"), ending with the formal proposal that neuronal mechanisms of attentional selection are indeed universal across modalities. Next we consider what factors/parameters might be feasible to compare across modalities (see "What can we measure across modalities to test universality?"). We then describe an example fMRI-based paradigm that might be so employed (see "An example paradigm"), and indicate how it can be extended to various modalities for comparison (see "Extending the ADD design to other modalities"). Finally, to provide some anatomical/neurophysiological context for the concepts introduced, we provide a brief discussion of relevant neuroanatomical mechanisms (see "Neuronal mechanisms"), and outline how the highlighted concepts relate to some previous work in the field (see "Relevance to preceding theories"). Overall the discussion is intended to be largely conceptual so as to stimulate interest in the question of universality and to foster new research that will broaden and deepen our understanding of the brain and its attention-related mechanisms.

## Extending attention-related concepts across modalities

**Fig. 1:**
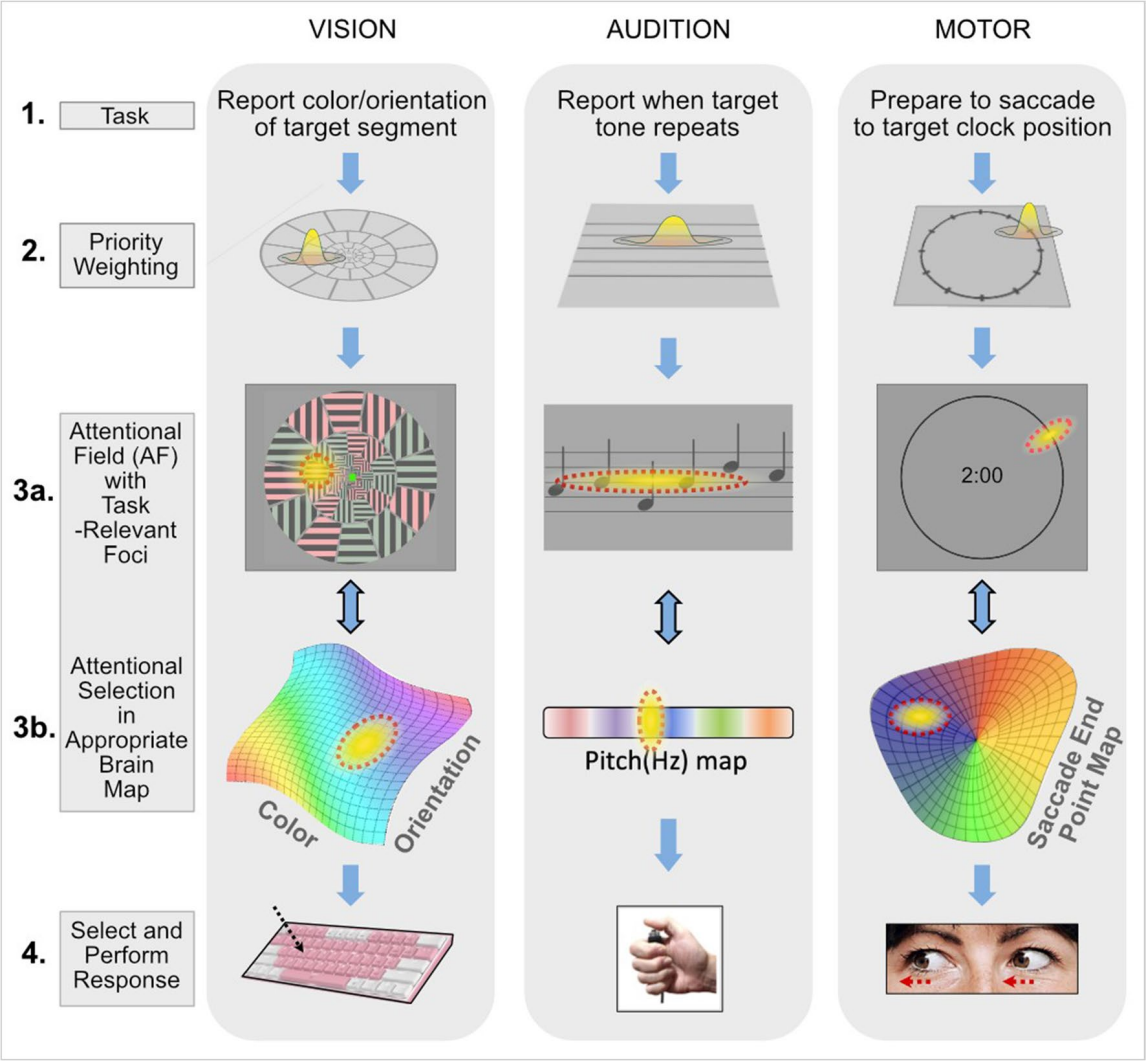
Conceptually similar operation of attentional selection in different modalities. Attentional demands of tasks in different modalities (row 1) generate information priority weightings (row 2), here shown as Gaussian functions expressing high priority as positive values. Priority weightings then generate a spatial pattern of attention, the attentional field (AF, yellow with dashed red outline) that can be referenced to the stimulus display (row 3a) but is applied to modality-specific neural maps in the brain (row 3b). Attentional enhancement of neural activity at a particular region in each map selects (enhances) the appropriate information, which is then used to select and perform an appropriate response (row 4)

First, let us consider how attention-related neuronal processes elucidated in the visual system might extend to other systems. As outlined in Fig. 1, simple tasks in the visual, auditory, and motor domains all require the generation of a high-level specification of the particular types of information required to perform each task (Fig. 1, row 1), which then results in a *priority weighting* of information potentially available in the display (Fig. 1, row 2). (How such a priority weighting is generated by the brain is beyond the current focus of this paper.) This priority weighting is then transformed into a spatial pattern of neural excitation/inhibition, the attentional field (AF), which can be referenced to the stimulus space (Fig. 1, row 3a), but is uniquely configured to the cortical space of a particular neural map (Fig. 1, row 3b) containing the appropriate, task-relevant information. Selective attentional enhancement of activity at an appropriate location within each map, potentially including suppression of neighboring regions, then permits output of the specific information required by action systems to select and perform the task-related response (Fig. 1, row 4). Our particular focus in the present context is both on the neurophysiological mechanisms of attentional selection (Fig. 1, row 3b) and on the proposal that such mechanisms share a common neural design across modalities.

**Fig. 2:**
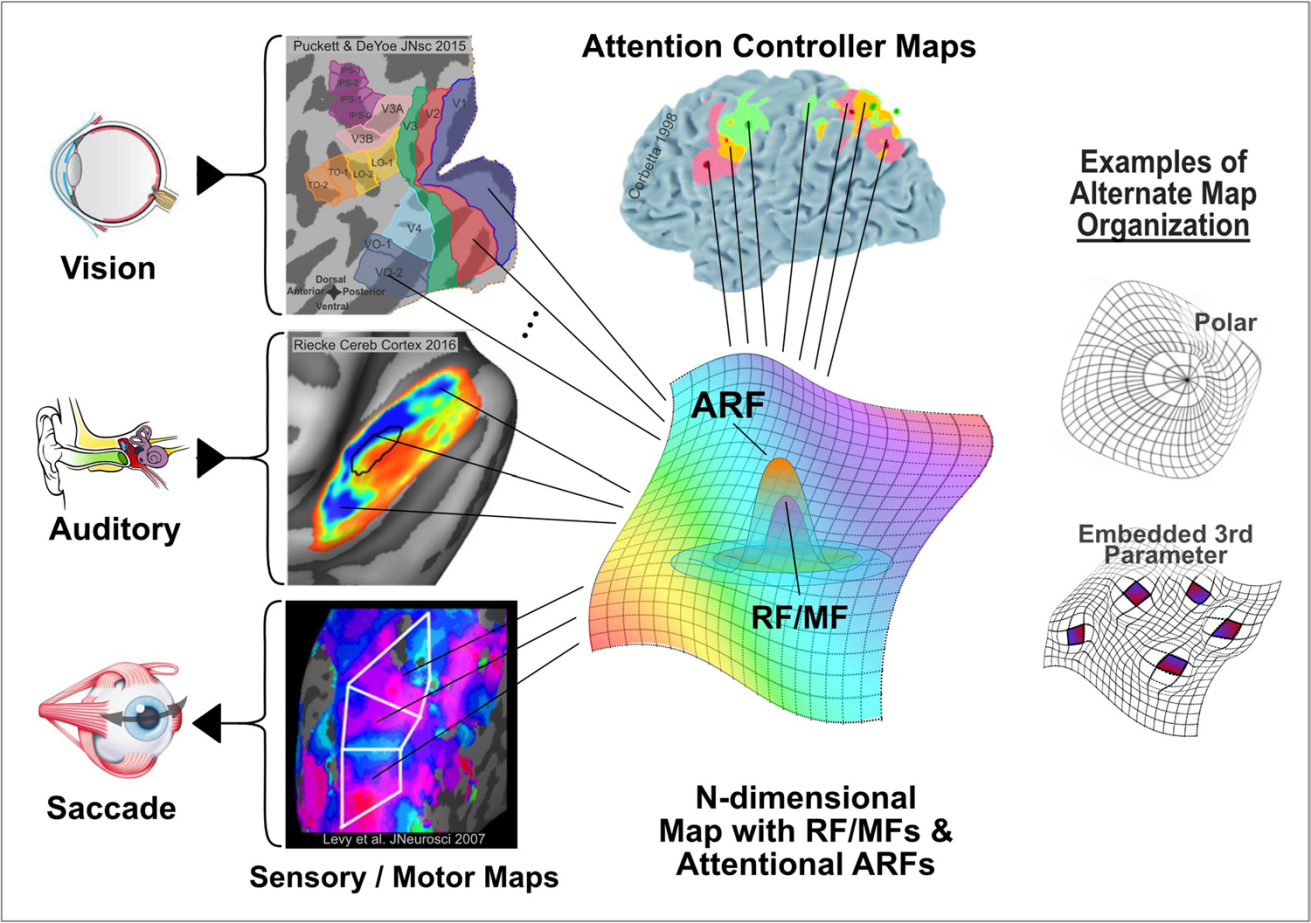
Modulation of neural maps may provide a universal mechanism of attentional selection. The neural map concept can be generalized for a variety of different cortical areas and representations of various parameters of interest (map colors, col. 3, center). Map organization can vary considerably (col. 2). In visual cortex, each individual area (colored patch, col. 2, row 1) contains its own map. In auditory and motor cortex (col. 2, rows 2,3) different areas (black, white outlines) may each contain a complete parameter map (e.g., tone frequency, saccade trajectory, indicated by map colors). The pattern of attentional modulation within a map is controlled by frontal and parietal areas (col. 3, top) that, themselves, may contain priority maps used to create the attentional pattern needed to select specific task-relevant information. Attentional selection of information encoded by receptive fields or motor fields (RF/MF) within a map is implemented via an interaction with attentional receptive fields (ARFs) that transform the pattern of attentional modulation applied to an area into a local modulatory signal (see Fig. 3 and associated text)

To begin to understand how the mechanisms of attentional selection may be universal, we must first generalize the notion of a "neural map" to encompass a variety of cortical representations of information within different functional areas and modalities (Fig. 2, column 2). In this context, we broaden the term "map" to refer to the organization by which a particular type of information "is mapped onto" (is represented by) the neurons of a particular brain area. In the visual system, this might be a retinotopic or orientation selectivity map. In audition, this might be a cochleotopic or audio-spatial map. In the saccadic motor system this could be a saccade endpoint or saccade trajectory map. Often this organization may be topographic; however, this is only one potential alternative. Other forms of organization may also be appropriate, especially for cortical areas representing more complex types of information (e.g., faces, body movements). Whatever the organizational form of information within a particular area, it must be available to the top-down, control mechanisms (Fig. 2, col. 3, top), which configure the pattern of attentional modulation that must be applied to a map in order to select the required, task-relevant information. (Note that this scenario might also apply to maps in other portions of the brain such as the colliculus and thalamic pulvinar, which are also involved in attentional control (Goldberg & Wurtz, 1972; Saalmann et al., 2012; Schneider & Kastner, 2009; Shipp, 2004).)

We stress that a neural "map" need not be conventionally topographic. For example, the cytochrome oxidase "puff" pattern in V1 and "thin stripe" pattern in V2 consist of circumscribed patches of cortex associated with color processing surrounded by cortical tissue representing other dimensions such as contour orientation or movement (DeYoe et al., 1995; DeYoe & Van Essen, 1985; Hendrickson, 1985; Livingstone & Hubel, 1988; Preuss et al., 1999) (e.g., Fig. 2, lower right). As another example of an unconventional map, Patel et al. (2014) describe in detail how the Lateral IntraParietal visual area (LIP) may have some topographic characteristics that are not necessarily complete nor continuous. They point out that "the evidence for an eccentricity axis perpendicular to the polar angle axis is weak, while evidence of a discontinuous foveal representation is much stronger." Additionally, "the edges of the topographic map in LIP do not appear to align with any areal boundaries." They make the important observation that: "...the absence of a [regular] topographic map in no way implies an absence of a complete representation of visual space" ...even though that representation may be disordered (Patel et. al., 2014, pp 6). In other words, strict, conventional topography is not an absolute prerequisite for "spatial" processing and this may be true for attentional selection as well. This is especially apropos if one allows "space" to be a parameter that does not necessarily represent external physical space... as is certainly the case in many (most) non-visual cortical areas such as the acoustic-frequency organization of primary auditory cortex (Fig. 2, column 2, row 2). These maps also need not be purely low-level, sensory representations but could also include higher-level characteristics such as visual object category (Uyar et al., 2016) or auditory object perceptual grouping (Gurariy et al., 2021, 2023). The key concept here is *not* whether all areas are topographic but, rather, how can the unique organization of information within each cortical area/neural map be specified... topographic or otherwise.

In this context, it is important to note that the performance of certain types of behavioral tasks may not require a "map" of information to select the appropriate task-relevant information. For example, cingulate cortex is thought to be involved in “conflict monitoring” or "error detection" (Botvinick et al., 2004; Carter et al., 1998; Orr & Hester, 2012), which may not require attentional selection of one unique parameter value from a continuously mapped range of similar values. Also, even if a cortical area contains a topographic map of some type, this organization may not be important for its contribution to some types of task. Moreover, not all of the information that happens to be available within a particular area is necessarily used for all computations that are performed there. For example, the Lateral Occipital Cortical area (LOC) is known to have a crude retinotopic organization (Uyar et al., 2016) due to broad pooling over its retinotopically organized inputs. However, this crude retinotopy may be "vestigial" and not functionally relevant for subsequent real-world, object-based, computations and representations. Our point here is to stress that, for attentional selection, the most functionally relevant features of a cortical map are the type of information represented there and how that information is organized, topographically or otherwise.

**Fig. 3:**
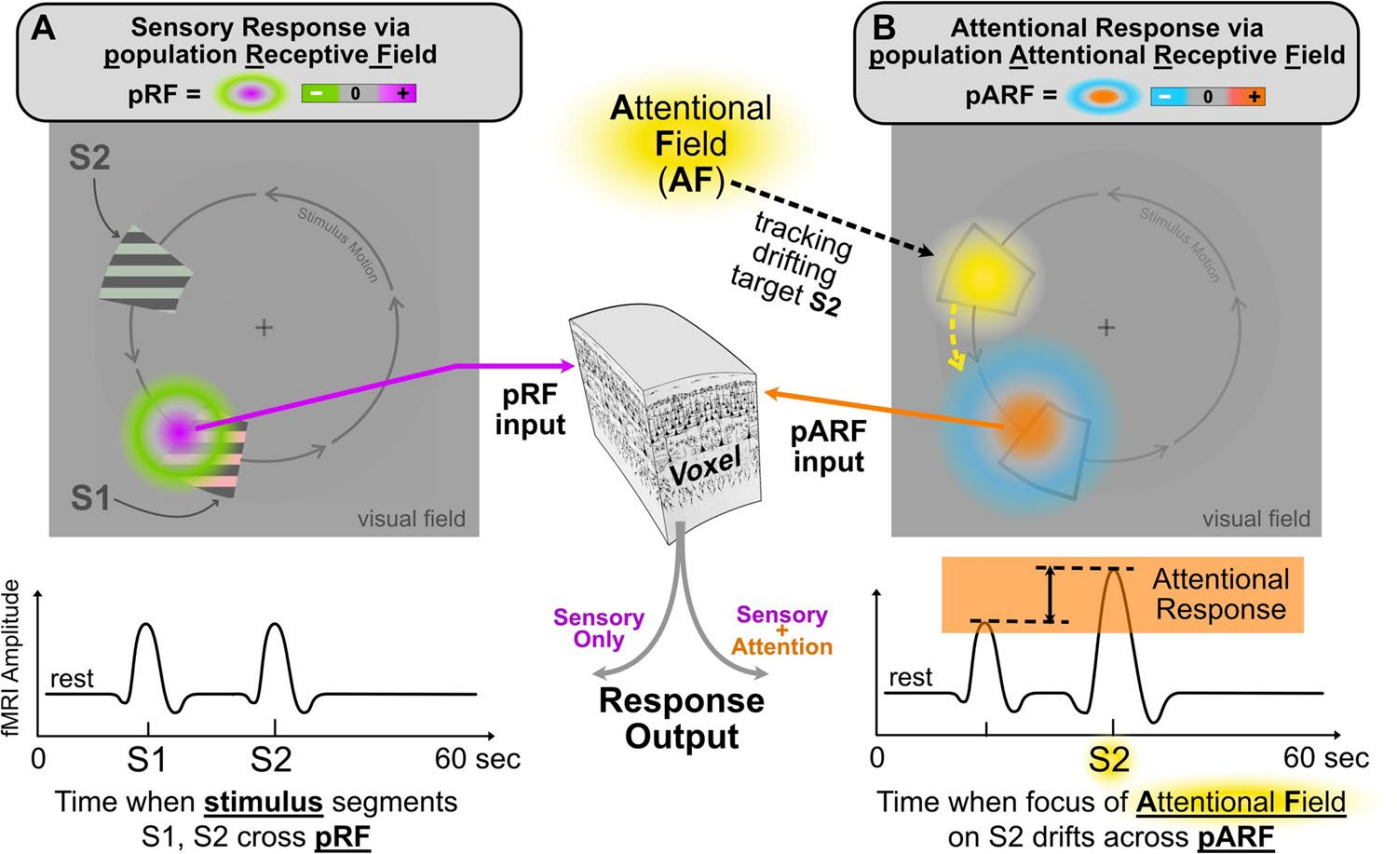
Example of pRF and pARF contributions to a voxel's response. The fMRI response of a voxel in visual cortex consists of 2 components: (**A**) Sensory and (**B**) Attentional. The left visual field diagram (**A**) depicts two stimuli (S1, S2) slowly rotating about a center gaze fixation marker (+). A voxel's population receptive field (**pRF**), represented schematically as a difference-of-Gaussians function (magenta, green colors), is shown at lower left of the visual field. As the stimuli S1, S2 drift across the fixed pRF, they each produce an fMRI response as illustrated in the sensory response waveform at lower left. The right visual field diagram (**B**) depicts an attentional field (**AF**, yellow) with a single focus directed to stimulus S2 (shown here as a gray outline for reference). The subject is tasked with maintaining gaze at the center fixation marker (+) while simultaneously attending to stimulus segment S2 as it slowly drifts around the fixation point. The voxel's population attentional receptive field (**pARF**) is shown schematically as a large difference-of-Gaussians function (orange, blue) that is fixed at a location roughly overlapping the pRF. (Note: diagrams A,B represent the same visual field but are shown separately for clarity.) As the focus of attention in the AF slowly drifts over the pARF, it generates the attentional response (orange box) consisting of an enhancement of the sensory response (via pRF) to stimulus S2. Note that the pARF is not responsive to the sensory stimulus itself, only to the attentional pattern. Note, the lower right response waveform is a *combination* of the sensory (A) and attentional (B) response components. (Figure of cortical voxel courtesy of https://neuwritesd.org/2014/07/10/cortical-columns/)

Given an understanding of the foregoing issues, we can then ask if the neural mechanisms of attentional selection reflect a universal neural design across all cortical maps or is each instance unique? Indeed, it is relatively straightforward to envision how a cortical pattern of attentional modulation impressed upon a neural map of information may provide a widely applicable, if not universal, mechanism of attentional selection. If one allows for the generalization of the neural map and associated attentional field concepts, then the generic application of an attentional field to a neural "map" might, indeed, provide a potentially universal mechanism for implementing attentional selection within almost any modality (Buschman & Kastner, 2015, pp. 140). The key, then, to understanding each modality-specific instantiation would seem to lie in (1) specifying the type and configuration of information represented within each neural map by receptive/motor fields of its constituent neurons (Fig. 2, RF/MF), (2) specifying the spatio-temporal pattern of the attentional field (AF; Fig. 1) and (3) specifying the mechanism by which the AF acts upon the neural map, herein termed the Attentional Receptive Field (ARF; Fig. 2). For example, the attentional selection of one specific sensory stimulus or action alternative from among many similar stimuli or movement options could involve impressing a stereotyped pattern of focal enhancement/suppression upon an appropriate map at an appropriate location (cf. Fig. 2, col. 3, center) (Cisek, 2007). The mechanism responsible for one’s ability to attend to your spouse’s face in a crowd and the ability to reach out to press the ‘Q’ key on your laptop could both employ a similar attentional mechanism applied to a task-relevant neural map whose content includes the representation of many similar faces or the representation of many similar hand trajectory endpoints.

While conceptually attractive, one must ask to what extent such a generic, universal mechanism is truly applicable across multiple modalities. Indeed, one can imagine scenarios in which a universal mechanism might *not* fully explain observed behavior. For instance, in vision, it is widely accepted that when a simple visual feature, (e.g., the color blue) is the focus of attention, then a group of objects that have the same blue color will evoke a perceptual "pop out" effect, even if the spatial distribution of those objects is irregular (Treisman & Gelade, 1980). In this way, multiple visual objects and their locations can be selected simultaneously by visual attention. In the saccadic motor system, however, multiple saccade trajectories could be planned, but there is a restriction on how many different saccade directions can be attentionally selected immediately prior to execution because the eyes can only saccade in a single direction at one time. Thus, the first task requires a multifocal (or spatially complex) attentional field, but the saccade task necessarily requires a single attentional focus. It is conceivable, then, that a less complex attentional mechanism may have evolved for saccades while a separate, more complex mechanism evolved for vision. While such issues remain to be explored more fully, our present purpose is simply to point out potential reasons to suppose that attention may not operate in precisely the same manner across modalities (Huddleston, Swanson et al., 2021b). Ultimately, a careful, detailed examination of potential differences in attentional mechanisms across modalities, and how such differences might affect behavior, will be key to determining the degree to which such mechanisms are truly universal throughout the brain.

Given the preceding perspective, it is important to point out that the potential existence of a universal neurobiological mechanism for attentional selection does not necessarily imply that the ultimate behavioral effects in each modality will be identical. For example, it is well known that the precision of attentional selection varies significantly both across the visual cortical hierarchy (Greenberg et al., 2012) and (with eccentricity) within the visual field (Intriligator & Cavanagh, 2001). One might suppose that this latter feature reflects the property of cortical magnification whereby similar-sized foci of attentional enhancement in the cortex will affect a smaller area of the central visual field than in the peripheral field (Qiu et al., 2006). However, detailed measurement of attentional crowding limits show that they scale much faster than predicted by either acuity or cortical magnification in V1 (Duncan & Boynton, 2003; Intriligator & Cavanagh, 2001) and are significantly different for the upper versus lower visual field (Intriligator & Cavanagh, 2001). Such detailed psychophysical data (especially the upper vs. lower field differences) led Intriligator and Cavanagh to suggest that such "attentional resolution" might be determined by parietal cortical areas rather than occipital visual areas, since the former, but not the latter, exhibit differences in upper versus lower field topography that are more consistent with the psychophysics (Fortenbaugh et al., 2015; Maunsell & Newsome, 1987). The point of this example is that the quantitative properties of both neural maps and attentional fields applied to them can affect behavior even though the neural mechanisms responsible for attentional selection may still reflect a common design. Untangling which factors may be responsible for apparent differences in attention-limited behaviors will be important for determining if the underlying neural mechanisms of selection are universal or not.

If we extend these insights from visual attention to other modalities such as the auditory system, it becomes apparent that detailed quantitative information about particular cochleotopic maps and their associated attentional fields are needed to permit a comparison with attentional mechanisms in the visual system and their corresponding behavioral effects. Similarly, in the saccadic motor system, one might expect that saccade endpoint maps may exhibit unique features such as differences in end-point resolution at different eccentricities and upper versus lower field locations. Do they have a property analogous to visual cortical magnification that varies substantially across visual areas (Harvey & Dumoulin, 2011), and how is the pattern of attentional modulation distributed across such a map?

Though the foregoing suggests that a variety of attention-related features might be compared across modalities, we next highlight a small subset that, at first blush, appear to share conceptual similarities across multiple sensory and motor systems. We also note, as have others (Douglas & Martin, 2004), that the brain, and particularly the cerebral cortex, evolved anatomical/physiological characteristics that, despite some variation across its surface, are arguably more uniform than disparate, at least in terms of the fundamental neural computations within each small patch of cortex. This too suggests that the cortical neural mechanisms for attentional selection might share a common design across modalities. Given this perspective, we propose the *testable hypothesis* that the neural mechanisms of attentional selection are universal across modalities even though the selected content may be specific to each modality. As a corollary, it then follows that apparent differences in attention-related perceptual effects across modalities will primarily reflect the different inputs (different sources of information to be processed and/or motor control signals) and output targets of each area, rather than major differences in the local neural mechanisms of attentional selection.

## What can we measure across modalities to test universality?

To practically compare neural mechanisms of attentional selection across modalities, we need an approach that (1) can be readily applied across modalities, (2) permits simultaneous comparison of neuron populations from many sites throughout the brain, and (3) can quantitatively measure key components of the attention selectivity mechanism. Given these criteria, functional neuroimaging (e.g., fMRI) currently appears to provide a particularly appropriate approach. In recent years, imaging voxel sizes have shrunk to the environs of 1 mm^3^ or slightly larger so that brain-imaging studies in humans and multi-neuron studies in animals have slowly converged toward a meso-scale focus that now provides a basis for comparing attentional mechanisms across modalities at an unprecedented level of detail and explanatory power. This modern meso-scale approach to human imaging arguably evolved from classical single neuron receptive field concepts (Hubel & Wiesel, 1962, 1968). Indeed, human studies by Dumoulin, Wandell, and others (Dumoulin & Wandell, 2008; Wandell & Winawer, 2015) put forward the notion of a population Receptive Field (pRF), which, echoing the concept of receptive fields of single neurons, can be defined as that portion of the visual field to which an imaging voxel is responsive. In other words, the pRF is the circumscribed region in visual space that causes the voxel to change its fMRI signal when a visual stimulus is drifted across or flashed within it. Figure 3, panel A, illustrates the concept of the pRF as it might appear delineated within a visual field containing two colored and striped stimuli (S1, S2) slowly drifting around a central fixation marker (+). The color/stripe patterns of the stimuli change randomly every 2 s so as to strongly activate the voxel. The pRF is here represented schematically as a typical difference of Gaussians (DOG) visual sensitivity function with an excitatory center (magenta) and an inhibitory surround (green). As each stimulus segment drifts across the pRF, it evokes an fMRI response as shown in the underlying timecourse plot. If the pRF and its interaction with the stimulus are computationally modeled, the response waveform can be predicted and used to iteratively refine the pRF model.

Since a 1 x 1 x 1 mm or slightly larger imaging voxel is typically composed of thousands of individual neurons, the pRF represents a composite of all the receptive fields of the entire responsive neural population within the voxel (including their variance in size and position). Although a voxel's signal reflects many individual neurons, it is important to note that the size of a modern voxel is on roughly the same scale as functional cortical "columns" and other features such as the cytochrome oxidase puffs and stripes in visual areas V1 and V2, respectively. At such a scale, neurons within a voxel/cortical column may be relatively homogeneous, so that, even at the population level, some aspects of selectivity are still apparent. For instance, imaging voxels are sufficiently small to easily (even preferentially) reveal organizational features such as cortical magnification of the foveal visual field and scaling of receptive field (pRF) sizes with eccentricity and across visual areas. Moreover, compelling evidence from Logothetis and collaborators (Logothetis, 2003; Logothetis & Wandell, 2004) indicates that blood oxygenation level dependent (BOLD) fMRI signals may preferentially reflect local field potentials rather than action potentials. This suggests that fMRI may be particularly appropriate for examining attention-related modulatory signals arising from fields of synaptic endings spread across whole cortical areas/maps. (*Note*: Throughout this paper we use the "p" prefix to denote voxel/population scale factors (e.g., pRF). When this prefix is not present, the term typically refers to a neuron-level factor (e.g., RF) or to a factor that is not necessarily method dependent (e.g., AF). Occasionally, we will use both (e.g., RF/pRF) when we wish to emphasize that a particular factor likely applies at both single-cell and population levels.)

Given the foregoing pRF perspective on human visual neurophysiology, the study of attention-related neural mechanisms in humans can also be approached on a similar scale of analysis (Klein et al., 2014; Puckett & DeYoe, 2015).^1^ For example, Fig. 3, panel B depicts the same visual field as in panel A but instead focuses on the attentional input to the voxel. The observer is asked to stare continuously at the central fixation marker (+) but simultaneously shift attention and track stimulus S2, repeatedly reporting its color/orientation by button press. To perform this task, attentional control centers in the observer's brain create an extended pattern of attentional modulation, an attentional field (AF), which, for this task, consists of a circumscribed focus (yellow) that drifts with target S2 as it moves around the display. Note that the complete attentional *field* covers all visual space even though its modulatory strength over much of that space might be effectively zero except in and around the location of the attended stimulus. The voxel of interest is phasically affected by the focus of attention when it slowly drifts across a circumscribed region of visual space, which we term the population Attentional Receptive Field (pARF).^1^ The pARF roughly overlaps the pRF but typically is much larger and has its own independent structure, represented here as a relatively large DOG with an enhancing center (orange) and suppressive surround (blue). The pARF is not itself responsive to the visual stimulus but, rather, is sensitive to the drifting attentional field. The effect of attention on the voxel via the pARF is then evident as an enhancement of the sensory response to the attended stimulus, S2 (Fig. 3B, underlying response waveform plot). The pARF makes the voxel sensitive to the spatial pattern of the attentional field in a manner analogous to the stimulus sensitivity conveyed by the pRF. (At the level of individual cells, both the RF and ARF may be coexisting properties of the same neuron(s), the first determining the response to the sensory inputs and the second determining the modulatory effect of the top-down attentional inputs.) A comprehensive understanding of a voxel's activity thus requires knowledge of all four factors: the visual stimulus and the pRF plus the attentional field and the pARF.

**Fig. 4:**
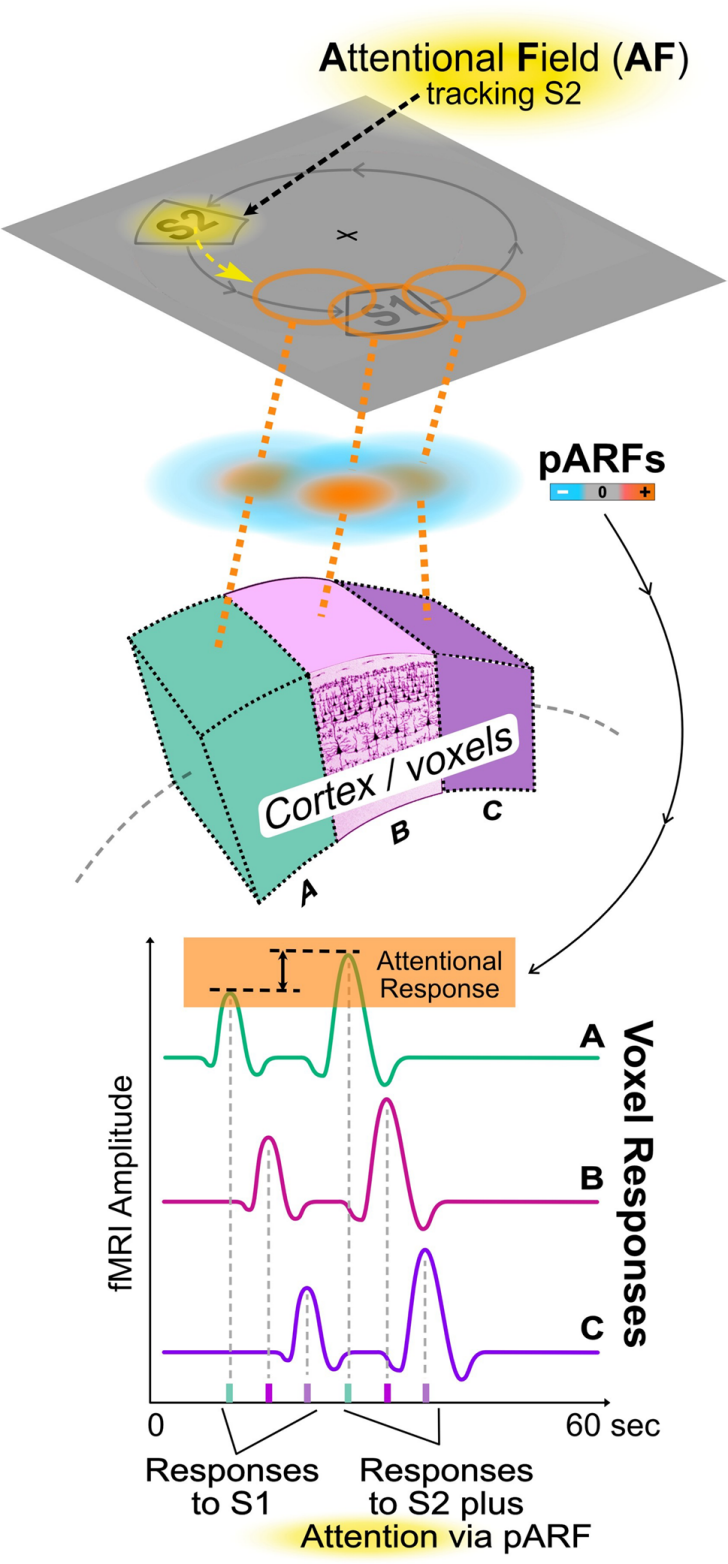
Population attentional receptive fields (pARFs) of different voxels are distributed in visual space. The attentional component of a voxel's fMRI response reflects the interaction between a pattern of attention and a voxel's pARF. The fixed visual field locations (and sizes) of pARFs associated with different voxels typically varies as illustrated in the upper portion of the figure (orange circles on visual field diagram and underlying difference of Gaussians functions; orange, blue). Due to the spatial offsets among the pRF's (not shown) and pARFs of the different voxels, their respective response waveforms (bottom of figure) will be delayed in time (and in peak width if the pARFs differ significantly in size)

Figure 4 portrays the pARFs for a small group of three neighboring voxels (A, B, C). As noted by Puckett and DeYoe (2015) the pARFs of adjacent voxels tend to vary somewhat in size and position though they are arranged roughly consistent with the overall visual field topography of the particular area of interest. For the voxels illustrated in Fig. 4, their pARFs happen to be distributed along the path traversed by the stimuli S1, S2 (gray outlines). Their respective spatial offsets along this path then cause a respective time shift in their responses to the drifting attentional focus (yellow) similar to the delays of the purely sensory responses to S1 via the voxels' pRFs.

## An example paradigm

Despite the seeming importance of addressing the potential universality of attentional mechanisms throughout the brain, there has been a shortage of theoretical concepts and methodological approaches that can be applied equally well across modalities. Single neuron methods have the disadvantage that comparisons across multiple modalities and numerous brain sites are generally infeasible due to the required time and small numbers of neurons sampled. Whole brain potentials can be recorded widely but lack sufficient specificity to compare individual cortical areas, much less different subpopulations within individual areas. At present, functional brain imaging (i.e. fMRI) may provide a way forward.

**Fig. 5:**
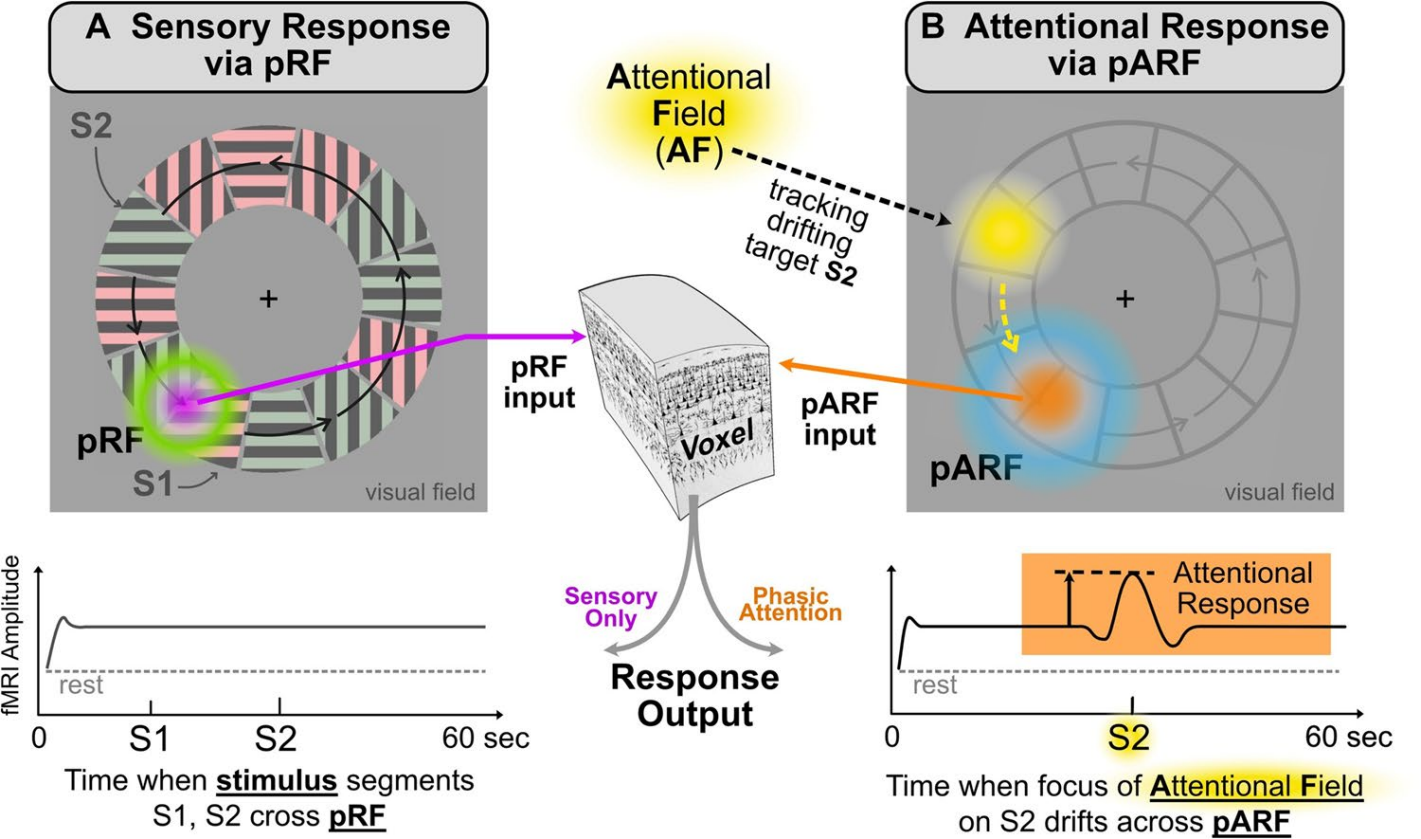
Isolating the attentional component of a voxel's response. One way to isolate and characterize a voxel's attentional response component is to use a stimulus consisting of a ring of closely spaced segments as illustrated in visual field diagram **A**. In this case, stimulus segments S1 and S2 do not evoke separate fMRI responses because the ring of segments causes a roughly constant activation of the pRF as illustrated in the response output plot at the lower left. As illustrated in visual field panel **B**, the subject is required to lock attention on stimulus segment S2 which creates an attentional field (yellow) that drifts with S2 as the whole stimulus array slowly rotates around the fixation point (+). (The subject must maintain gaze fixation at the center while covertly attending to the eccentric stimulus segment S2.) As the focus of attention (yellow) slowly drifts across the population attentional receptive field (pARF, orange/blue), it generates the isolated attentional response component which appears as a modulation of the ongoing, constant sensory activation (orange box). Figure of cortical voxel courtesy of https://neuwritesd.org/2014/07/10/cortical-columns/

To perform a cross-modal comparison of attentional mechanisms at the population level, an initial step is to empirically measure the population attentional receptive fields (pARFs) of multiple voxels within cortical areas containing information relevant to an attention-dependent, experimental task; one that can be employed across multiple modalities. To accomplish this in quantitative detail, Puckett & DeYoe (2015)^1^ developed a useful experimental strategy analogous to using a drifting visual stimulus to map pRFs in visual cortex. Rather than drift a discrete visual stimulus through the field of view, a unique task and display are used to induce the subject to drift their focus of attention through the visual field while the visual stimulus features remain dynamically uniform for local pRFs. Figure 5, panel A, shows an example of this Attentional Drift Design (ADD) using a stimulus consisting of an array of contiguous, randomly colored and striped segments. Every 2 s, the pattern of each segment is re-randomized and the whole array slowly rotates at 1 revolution per minute, so that, over time, any given location experiences each of the color/orientation patterns equally. For a voxel whose pRF (magenta/green) is located along the stimulus path, this visual stimulus generates a roughly uniform level of neuronal activation as illustrated in the underlying timecourse plot of Fig. 5A. However, the observer is then instructed to constantly fixate the "+" at the center of the display while simultaneously attending to S2 and repeatedly reporting its color/orientation pattern by button press. As depicted schematically in Fig. 5, panel B, this task forces the subject to create a focus of attention (yellow) that drifts along with S2 and the whole stimulus array (dim gray outlines in panel B). As the focus of attention drifts across a voxel's pARF (orange/blue), its ongoing sensory response is phasically modulated, thereby allowing the attention-related response to be isolated and measured directly (orange box in response waveform plot). The stimulus and task are specifically designed to force the observer to constrict the focus of attention to its smallest possible size (for the tested eccentricity). If the pRF has been measured previously (Fig. 3A) and the focus of attention is assumed to be a Gaussian or similar function under these task conditions, then the attentional response waveform can be accurately predicted and compared to an empirical waveform through iterative computational modeling and optimization of the size and shape of the pARF. The complete computational model includes the stimulus configuration, its interaction with the pRF, the complete pattern of the AF, its interaction with the pARF and the interaction between the pARF and pRF, all of which together determine the complete output signal of a voxel (Puckett & DeYoe, 2015).

Note that the stimulus array shown in Fig. 5 is experimentally adjusted in density for each individual subject so that only through focused attention on the cued target segment can they correctly report its moment-by-moment, color/orientation pattern without interference from neighboring segments. This forces the subject to restrict their window of attention to be as small as possible. (This minimum size varies with the eccentricity of the ring containing the attended segment (Intriligator & Cavanagh, 2001)). Additionally, the task of reporting the dual parameter conjunction of color and orientation is known to require, top-down, focused attention (Treisman & Gelade, 1980), further ensuring that the subject's task performance is under constant attentional control. Under these conditions, several dozen voxels having pRFs distributed along the ring containing the attended stimulus segment can provide multiple pARF estimates for each visual area of interest. Puckett and DeYoe reported that for these conditions, the pARF was well modeled as a difference-of-Gaussians function with a suppressive surround (Fig. 5B, orange/blue). (Note: In Puckett & DeYoe (2015) the pARF is not explicitly specified as a separate neural mechanism but is implicitly included in the model component that they termed the AF.)

The preceding example was intentionally aimed at forcing the subject to carefully control and minimize the size of their attentional focus. However, once the pARF has been estimated precisely with the AF under this tight control, more complex AF distributions can be employed and actually measured using the same modeling approach but with the iterative optimization focused on the AF profile rather than the pARF. In this way, the potentially unique AFs created by different subjects can be measured and compared. An example of a more complex AF distribution can be easily imagined using the stimulus array of Fig. 5 but requiring the observer to repeatedly report if the colors of *two* cued stimulus segments (S1, S2) are the same or different as their patterns change moment by moment. In this case, attention must be allocated more or less simultaneously to the two separate segments as they slowly rotate with the whole array. As each of the two attended locations passes over the pARF, the isolated attentional response will now have two peaks rather than just one (not illustrated in Fig. 5). Again, if the pARF has been previously estimated, then the actual spatial pattern of a more complex AF can be extracted from the timecourse of each voxel's response.

Note that, in general, each cortical area/neural map may have its own unique AF distribution depending on the types of visual information represented there and the types of information targeted for attentional selection. In areas that don't represent information currently targeted by attention, the attentional field may be effectively uniform and suppressive, as suggested by the biased competition model of attention (Desimone & Duncan, 1995). We propose that the attentional field within a particular cortical area (or areas) that most strongly represents the information relevant to the current task determines (by winner-take-all) the overall behavioral effect of attention (e.g., reporting the color/orientation of a specific visual target segment).

## Extending the Attentional Drift Design (ADD) design to other modalities

The ADD described above can be extended for use with a variety of attentional tasks and modalities. For example, one can readily conceive of a design in which the observer's attention slowly drifts through some non-spatial featural dimension such as color or morphing faces. The key is creating constraints that cause the subject to exert consistent control over their allocation of attention within the parameter space of interest. Moreover, this approach can be extended to other modalities (Dick et al., 2017; Puckett et al., 2017). Audition provides an example of attention drifting over the frequency (pitch) range of a repeated stimulus tone. As illustrated in the center column of Fig. 1 (row 3), a series of random 1-s pure tones can be presented successively with the observer cued to attend to the middle frequency/pitch in the sequence. When the observer detects the cued tone, the task then requires them to shift attention to the next higher frequency, for example. The attentional shifts occur relatively slowly (e.g., every 5 s), so that over 40 s the observer has attended to each of eight different tone frequencies in the random sequence. (Note that, on average, the random stimulus sequence presents all eight tones in the sequence within 8 s, so that, over time, the stimulus evoked activity of all voxels in the map is roughly equal, except when modulated by attention.) Within a task-relevant cochleotopic brain map, a voxel tuned to a particular frequency (its population frequency receptive field, pFF) will be modulated by attention whenever the attended pitch is within its preferred frequency range. As with the visual ADD paradigm, an estimate of the profile of the auditory population attentional receptive field (pARF) can be extracted from the fMRI timecourse of voxels tuned to particular frequencies. Strictly speaking, since a voxel may be tuned to a range of frequencies, its auditory pRF (pFF) first needs to be determined using conventional frequency mapping stimuli. Then, using the ADD paradigm, the auditory pARF is iteratively modeled to predict the empirical fMRI waveform. Again, once the pARF is estimated using a tightly confined attentional pattern, then the modeling can be used to measure and explore more complex attentional fields and tasks.

In addition to using this design with sensory modalities, this ADD approach also can be readily extended to the motor domain (Huddleston, Catterson et al., 2021a). A simple visual display can be used that consists of a circle at a fixed radius from a central, rapid serial visual presentation (RSVP) stream (Fig. 1, row 3a, right). The RSVP stream consists of random letters flashed every 4 s interspersed with cued target locations (e.g., 2:00) to which the subject *might* have to saccade. Infrequently (randomly, on average every 40 s), a GO cue is presented signaling to the subject to actually saccade to the most recently cued location. This task effectively confines visual attention centrally to monitor the RSVP stream while motor attention is directed peripherally to successively cued clock positions. The infrequent, randomly timed saccades have little effect on the averaged fMRI timecourse. In this way, motor attention is slowly swept around the circle allowing the motor pARF to be modeled and mapped across various brain regions in frontal and parietal cortex.

The foregoing examples highlight the key features of the ADD approach. They each involve a task that requires the observer to drift their focus of attention through a relevant parameter space (e.g., visual field, tone frequency, motor target) represented within some cortical (or even subcortical) area of interest. As the focus of attention passes over the pARFs of voxels within each area, they are temporally modulated, and the resulting waveform will reflect the unique pattern of the AF within each area. The primary advantage of this approach in the current context is that it allows quantitative estimation of the pARF and the attentional field (AF) within and across modalities so that they can be compared and used to assess their potential universality. However, we stress that the ADD paradigm presented here is just one example of an approach that can be used to advantage across modalities. There are undoubtedly others!

## pARF/pRF interaction

In the framework presented above, we have intentionally avoided a detailed discussion of the nature of the interaction between a voxel's pARF and its pRF, though we suggest that, at each time point, a simple spatial multiplication may be a reasonable first approximation. Indeed, there is a voluminous literature on the effects of attention upon neuronal responses and pRFs summarized in several previous articles (Anton-Erxleben & Carrasco, 2013; Buschman & Kastner, 2015). Some of the documented neural effects of attention include increased sensitivity/gain to stimuli (Kay et al., 2015; McAdams & Maunsell, 1999; Motter, 1993; Reynolds & Chelazzi, 2004; Reynolds et al., 2000; Sprague & Serences, 2013), shifts in pRF/RF location and/or size (Baruch & Yeshurun, 2014; Connor et al., 1996; Klein et al., 2014, 2016, 2018; Moran & Desimone, 1985; Niebergall et al., 2011; Sheremata & Silver, 2015; van Es et al., 2018; Vo et al., 2017; Womelsdorf et al., 2006, 2008), reduced noise correlations (Cohen & Maunsell, 2009), increased synchrony (Fries et al., 2001), resolution of competition among multiple targets (Reynolds et al., 1999), and changes in multineuron population codes (Buschman & Kastner, 2015; Cohen & Maunsell, 2009; Mitchell et al., 2009), including shifts in category tuning toward an attended category (Cukur et al., 2013). However, there has often been an inadequate or simplistic characterization of the complete attentional field and how its effects are impressed upon whole areas/maps and their constituent neurons. For instance, it is not uncommon to assume that the AF is appropriately specified by the description of the attentional task and stimulus. However, when subjects are asked to direct attention to a single stimulus or an array of widely spaced stimuli, they may successfully accomplish the task using an attentional focus that can vary widely in shape, size, and position, thus leaving the AF poorly controlled. As mentioned previously, the use of multiple, closely spaced stimuli surrounding an attended target at the "attentional resolution limit" (Intriligator & Cavanagh, 2001) is one way to force the attentional focus to be positioned precisely on a target and contracted to a minimum size, thereby providing much greater experimental control over the attentional field. However, more complex attentional configurations also need to be considered. When attention is directed to a spatially well-defined object (even a simple rectangle), the attentional effects may appear to be confined within the object, thereby becoming distributed as a function of the shape of the object (Egly et al., 1994). On the other hand, feature-based attention directed to all the red objects in an array can simultaneously enhance responses of multiple cells/voxels associated with ARFs/pARFs aligned with each of the red targets throughout the display (Saenz et al., 2003). This suggests a particularly complex AF with both enhancing and suppressive regions distributed throughout the field of view. Indeed, one widely reported effect of attention on single neuron and voxel-based receptive fields is an apparent shift of the RF/pRF toward the location of a focus of attention (Baruch & Yeshurun, 2014; Connor et al., 1996; Klein et al., 2014, 2016, 2018; Sheremata & Silver, 2015; van Es et al., 2018; Vo et al., 2017; Womelsdorf et al., 2006, 2008). At first this may seem to involve a complex manipulation of the spatial characteristics of the pRF. Yet, such effects are readily anticipated if one considers the multiplication of a pRF with a pARF having a simple Gaussian enhancement profile offset from the center of the pRF (Klein et al., 2014). Whether such a simple scenario can quantitatively account for the observed empirical effects in more complex situations remains to be tested fully. However, the important point is that the experimental control of the complete AF with characterization of ARFs/pARFs is essential to understand how attentional and sensory inputs combine to determine the responses of single neurons and imaging voxels.

## Neuronal mechanisms

**Fig. 6:**
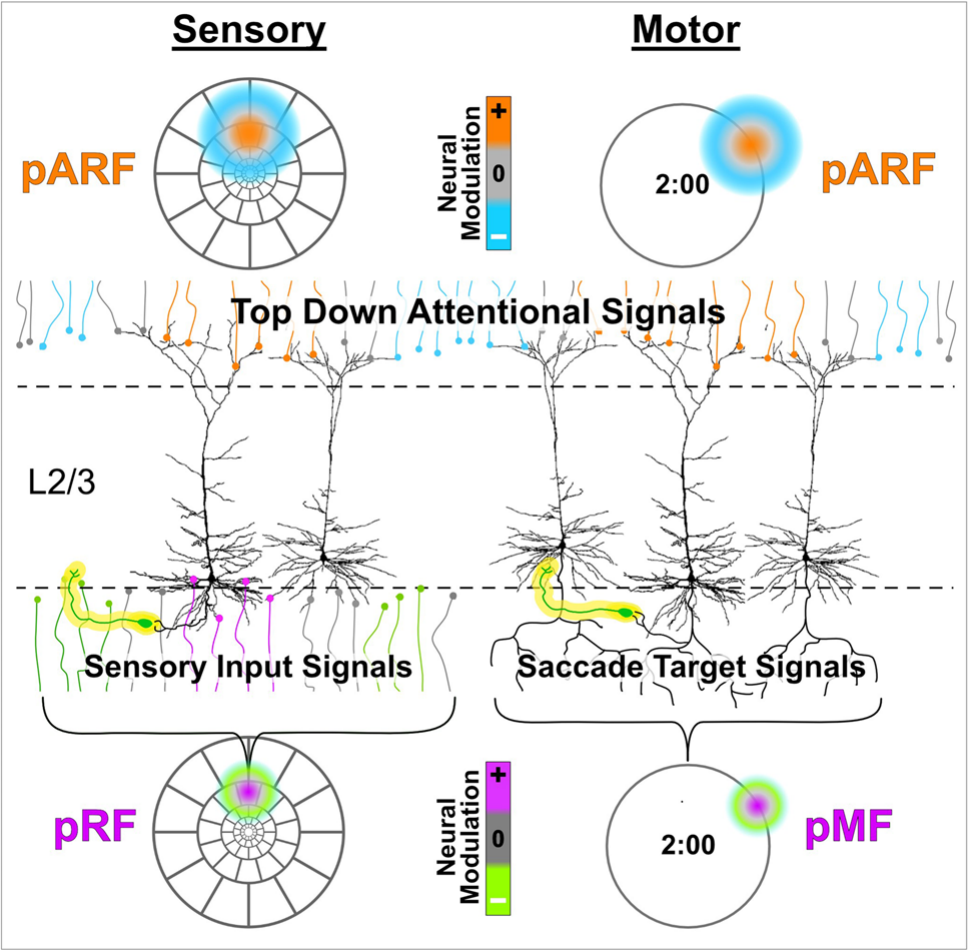
Speculative neuroanatomical correlates of a voxel's pARF and pRF or pMF: Sensory input signals (e.g., visual) representing target features or motor output signals (e.g., saccade trajectories) are distributed across fields of synaptic endings or axon terminations (under lower dashed line) that are topographically organized. Similarly top-down attentional modulation signals are distributed across fields of synaptic terminals (above upper dashed line) typically arising from fronto-parietal cortical areas. Enhancing vs. suppressing neural modulation indicated by color codes (center column). Ubiquitous inhibitory interneurons highlighted in yellow cause mutual suppression alleviated by focal attentional enhancement (Desimone, 1998). pARFs, pRFs, and pMFs are displayed as they would appear projected into the visual or motor target field. pARF = population attentional receptive field, pRF = population receptive field, pMF = population motor (end point or trajectory) field. Gray outlines in the sensory column represent stimulus array as in Fig. 3. Motor column large gray circles represent array of possible saccade end points. L2/3 indicates cortical layers 2/3 where pyramidal output neurons typically reside

To help provide a more concrete view of these concepts, Fig. 6 schematically outlines a speculative relationship between a voxel's pRF and its pARF at the neuronal level. (Fig. 6 is not strictly anatomically accurate and is highly simplified.) Sensory input signals (e.g., visual, auditory) or motor output signals (e.g., saccade target) are distributed across fields of synaptic endings (axon terminations) that are, often, topographically organized "maps," as illustrated in Fig. 2 (col. 2). For example, the retinal sensory input to primary visual cortex is via the well-known pathway through the lateral geniculate nucleus of the thalamus that terminates in cortical layer 4 (Fig. 6, small colored dots with tails below L2/3). The input signals are then locally relayed by interneurons (not shown) onto basal dendrites of pyramidal cells of layers 2/3. By spatially sampling from this field of input signals, the pyramidal cells build receptive fields that, for an fMRI voxel, collectively represent the pRF. In the eye-movement domain, an analogous output scenario (Fig. 6, right) might arise from layer 2/3 pyramidal cells in frontal eye fields and/or parietal cortex, whose output signals represent a saccade target location or trajectory. The corresponding population motor field (pMF) for a particular voxel might then represent a saccade end point at the 2:00 position (Fig. 6, lower right). The output signals of such neurons are attentionally modulated by top-down signals that typically arise from fronto-parietal cortical areas (Felleman & Van Essen, 1991) but terminate in fields of synaptic terminals spread throughout superficial layers of the cortex (Fig. 6, colored dots with tails above L2/3). This field of modulatory inputs is then sampled by the attentional receptive fields of neurons within a voxel (collectively comprising the pARF). The pARF effectively transduces the incoming attentional pattern into moment-by-moment enhancement/suppression of the output signals of the neurons within the voxel thereby selecting (or suppressing) the task-relevant information represented there. In accordance with the concept of biased competition, the output neurons are also embedded in a mesh of widespread lateral inhibitory influences, which are represented schematically in Fig. 6 by the yellow highlighted inhibitory interneurons (again, highly simplified). Accordingly, the field of attentional modulatory inputs then provides the "bias" that breaks the mutual inhibitory competition, thereby allowing output of the locally represented information.

One final point noted by Puckett and DeYoe (2015) is that a voxel's pARF is generally much larger than its pRF (roughly 1.5–3X radius) with a suppressive surround that is even larger (not well represented in Fig. 6). This difference in size suggests that the pARF reflects a neural mechanism that is distinct from that of the pRF and has its own spatial properties, as might be inferred from the different laminar distributions (and sources) of synaptic endings associated with pRFs versus pARFs. Consequently, modeling the top-down modulatory effects of attention as a simple point-like gain control of the pRF may not be appropriate when complex tasks and associated attentional fields are involved.

## Relevance to preceding theories

As outlined above, our conceptual framework includes several components that can be compared across modalities to help determine if attention is mediated by a universal neurobiological mechanism. Specifically, these are: (1) neural "maps" and their constituent pRFs, (2) attentional fields (AFs), (3) population attentional receptive fields (pARFs), and (4) the interactions of pRFs and pARFs. Above, we have outlined our expanded concept of a neural map to include a variety of neural representations of information or action alternatives. Indeed, conventional spatial or featural maps are components of a number of influential theories of attention including Treisman's feature integration theory (Treisman & Gelade, 1980), Desimone and Duncan's biased competition theory (Desimone & Duncan, 1995; Kastner et al., 1998), and Reynolds and Heeger's normalization theory (Reynolds & Heeger, 2009), to mention a few. Whether a map is conventional, as in a retinotopic map, or more complex as in a "map" of facial features (Chang & Tsao, 2017), a key functional aspect is that the map is a representation of information extending across an array of neurons that can be modified by a pattern of modulation applied to it. Indeed, the "map," as we have broadly defined it, might also be seen as a population of neurons constituting a multivoxel pattern representation of some task-relevant parameter or parameters (Haxby, 2012; Haxby et al., 2001). In such a case, the attentional field (AF) represents the pattern of attentional modulation applied to the whole multivoxel pattern representation.

Our rationale for using the term "attentional field" is in accord with the idea that such a top-down modulating influence likely reflects the wide distribution of synaptic inputs from "higher" cortical areas in the parietal and frontal lobes on to superficial (and possibly deep) layers of "lower" cortical areas (Felleman & Van Essen, 1991; Paneri & Gregoriou, 2017). This distribution is generally consistent with the laminar "feedback" pattern identified by Felleman and VanEssen (1991). Our neuronal concept of an attentional field appears similar to Desimone and Duncan's "attentional template," which is described as a top-down bias on visual processing (Desimone & Duncan, 1995). The attentional field as envisioned in our framework is a conceptually separate entity that extends in space and time across an ensemble of neurons representing a neural map. This macroscopic conceptualization of an attentional field has often been absent (or possibly implicit) in earlier theories focused on single neurons. For example, in the work of Lee and Maunsell (2010) attention is more simply represented as a "response gain control" that modulates the effects of a stimulus on the firing rate of a neuron. Alternately, it may operate as a contrast gain modulation (Reynolds et al., 2000), or perhaps other configurations (Fiebelkorn & Kastner, 2020; Lee & Maunsell, 2010). In such theories, the more global, spatial pattern of this gain control across an entire visual area and its ensemble of neurons is often not addressed. Consequently, this can leave the spatial effects of attention to be "inherited" as a modulation of the spatial pattern of excitatory and inhibitory regions of a neuron's receptive field. This does not then allow for any independent spatial effects that, theoretically, could arise from the properties of the AF itself and the pARFs associated with different areas. More complex stimuli and attentional tasks may require the AF to have a more complicated spatial and featural profile with multiple or extended foci of enhancement and suppression. Both imaging and behavioral evidence for this sort of complex, non-contiguous attentional selection has been outlined in the past (Gobell et al., 2004; McMains & Somers, 2004).

A conceptualization of the AF that appears fairly similar to our framework was included in Reynolds and Heeger's normalization theory of attention (Reynolds & Heeger, 2009). In their model, the AF represents a multiplicative (gain control) pattern of modulation extending across a neural space (map) whose dimensions are pRF center location (within the field of view) and preferred orientation (within a featural orientation map; their Fig. 1). To simplify their exposition, the center location was expressed in only one dimension as was orientation preference. The spatial pattern of the AF appeared to be a Gaussian that was selective in orientation space. A key feature of the Reynolds and Heeger model is normalization, which, as a component of an attentional model, constitutes a more sophisticated mode of interaction between the AF and a neural map. Normalization is accomplished through the incorporation of an additional divisive "suppressive field" that is also defined within the same space as the attentional field but involves a relatively broad integration across both the pRF location and orientation dimensions. Their model AF enhanced a selected range of orientations but did not itself include explicit suppressive effects. However, the authors did mention that this could be added, if warranted. In the Reynolds and Heeger model, any attention-related suppressive effects are mediated by amplification of the stimulus drive field in regions that become included within the divisive suppressive drive field. Whether this is sufficient to account for all attention-related suppressive effects, especially as a model for attention within other modalities, remains to be tested experimentally. Indeed this may be a particularly informative focus for comparison across modalities. Reynolds and Heeger mention that their model may provide a more explicit mechanism that extends Duncan and Desimone's biased competition model of attentional selection (see Reynolds et al., 1999). The ubiquitous mutual suppression of biased competition presumably is the counterpart of the widespread suppressive field of the normalization model. Both models are sufficiently generic to be plausibly applied to other modalities. Moreover, Reynolds and Heeger go on to show how normalization could at least qualitatively account for a number of attention-related effects such as response versus contrast gain effects as well as scaling of neuronal tuning curves, all of which may or may not be similar across modalities.

In the present discussion, we have not explicitly included normalization or biased competition within our framework, though their inclusion would be an obvious way to extend our AF and ARF concepts. Exactly how the spatial characteristics of a complex AF would interact with a normalizing suppressive field would appear to be potentially complex, but amenable to modeling if the interactions remain linear over some reasonable range. Moreover, such additional complexity increases the likelihood that such a model may not be universally appropriate across modalities. Indeed, it would be most interesting to determine how and why any of the different models might vary in a substantial manner across modalities. If they do, it may help to indicate how neural processing and attentional selection may have been optimized for each modality.

In this paper, we have intentionally focused on attentional phenomena that tend to reflect top-down (Corbetta & Shulman, 2002; Desimone & Duncan, 1995; Kastner & Ungerleider, 2000), goal-directed (Corbetta & Shulman, 2002), endogenous (Berger et al., 2005; Posner, 1980; Theeuwes, 1991) attentional control mechanisms. In other words, attentional behaviors under the observer's volitional control, whether cued or purely voluntary (Gmeindl et al., 2016). Our emphasis was on the specific role of attention and how it operates within the context of tasks having a relatively simple, well-defined goal. Factors beyond our scope included the mechanisms by which the observer determines the goal of a task, determines what information is needed to perform the task, and performs a response if one is required. We also have ignored exogenously controlled attention since its properties appear to be notably different (Li et al., 2021). We focused primarily on the subprocess of attentional selection/enhancement and did not specifically address other subprocesses such as alerting (Posner, 2012), baseline shifting (Kastner et al., 1999; Seydell-Greenwald et al., 2014), reductions in low-frequency variability (Mitchell et al., 2009), slow fluctuations (Bressler et al., 2020), engagement/disengagement (Buschman & Kastner, 2015), or motivation (Engelmann et al., 2009). Within this restricted context, we assume that the primary role of attention is the selection, enhancement, and/or suppression of information or action alternatives. Accordingly, we also did not address issues such as attentional effects on "binding" and decision making. Finally, our ultimate focus was on neural mechanisms. Thus, our primary goal was to compare attention-related neural mechanisms across functional modalities (specifically vision, audition, and motor systems) with an emphasis on cortical mechanisms responsible for attentional selection within consciously performed behaviors.

## Conclusion

While our understanding of the brain mechanisms responsible for attention and its effects on behavior have relied heavily on experimentation and theories associated with vision, it is often tacitly assumed that these theories and mechanisms apply equally well to other sensory and motor modalities, even though there are potentially valid reasons to suspect that this may not be the case. Examining the available data on this issue can be difficult since the behavioral effects of attention in different modalities often seem disparate, and attempts by the same investigator to use identical methods across multiple modalities are rare. From a neuronal standpoint, however, one is struck by the overall similarity of basic neuronal anatomy and physiology throughout the cerebral cortex except for a few notable exceptions whose relevance to attention, if any, remains obscure. We propose to make this assumption explicit and hypothesize that the variety of behavioral effects attributable to attention within different modalities, and their associated cortical areas, reflects the different inputs (different sources of information to be processed and/or control signals) and output destinations rather than major differences in the effects of attention upon each cortical area. In other words, we propose that the neuronal effects of attention are ubiquitous and universal throughout the cerebral cortex. To begin to test this hypothesis we have outlined a useful experimental framework and fMRI methodology that can be applied to multiple modalities including vision, audition, and the saccadic motor system, in particular. Though the approach initially focuses relatively narrowly on comparing neural modulation/selection quantitatively across modalities, it is hoped that this will motivate future efforts to compare additional aspects of attention across a wide variety of modalities and submodalities throughout the human brain. This may then lead to a firm experimental basis for asserting a universal neural theory of attention or may lead to a deeper understanding of how attention has been specialized to utilize each modality to greatest advantage.

## Data Availability

Review article, not applicable.

## Footnotes

1 The nomenclature and model presented here is modified from that described in Puckett & DeYoe, 2015. In Puckett, for any moment in time, a spatially restricted patch of the attentional field directly interacts with, and is determined by, the location and extent of a voxel's pRF. In the model presented here, the patch of AF that affects a voxel is determined by the location and extent of the pARF which typically is somewhat different (larger and often offset) compared to the pRF (cf. Fig. 3).

## Code availability

Custom Matlab software for computation of population attentional receptive fields available upon request from E. DeYoe.
